# Retinopathy of Prematurity and Risk of Structural Brain Abnormalities on Magnetic Resonance Imaging at Term Among Infants Born at Less Than or Equal to 32 Weeks of Gestation

**DOI:** 10.1016/j.jpeds.2025.114711

**Published:** 2025-06-27

**Authors:** Shalini Roy, Laura Peterson, Beth Kline-Fath, Nehal A. Parikh

**Affiliations:** 1 The Perinatal Institute, Cincinnati Children’s Hospital Medical Center, Cincinnati, OH;; 2 University of Cincinnati College of Medicine, Cincinnati, OH;; 3 Neurodevelopmental Disorders Prevention Center, Cincinnati Children’s Hospital Medical Center, Cincinnati, OH;; 4 Department of Radiology, Cincinnati Children’s Hospital Medical Center, Cincinnati, OH;; 5 Department of Radiology, University of Cincinnati College of Medicine, Cincinnati, OH

## Abstract

**Objective:**

To determine if retinopathy of prematurity (ROP) is associated with brain abnormalities on structural magnetic resonance imaging at term-equivalent age in infants born preterm.

**Study design:**

We prospectively recruited 395 infants born preterm born at ≤32 weeks of gestation from 5 regional Cincinnati neonatal intensive care units. Eligible infants underwent routine ROP screening and diagnosis per international screening guidelines at 31 weeks postmenstrual age or 5–6 weeks of age (whichever came later). We obtained nonsedated structural magnetic resonance imaging at 39–44 weeks postmenstrual age. Brain injury/ maturational abnormality was quantified using the validated Kidokoro global brain abnormality score by a single neuroradiologist. We performed multiple linear regressions to determine the association between ROP and brain abnormality score while adjusting for several known confounders.

**Results:**

Of the regional sample of 395 infants born preterm, 134 (33.9%) developed ROP. Among those infants with ROP, 19 (14.2%) developed severe ROP (stage 3 or requiring treatment). In multiple linear regression analyses, ROP (any severity) (*β* = 1.5 [95% CI: 0.3, 2.8]) and severe ROP (*β* = 2.3 [0.1, 4.5]) remained significantly associated with global brain abnormality score, independent of multiple confounders. In secondary analyses, ROP was significantly associated with cerebellar and deep nuclear gray matter but not white matter or cortical gray matter abnormalities.

**Conclusions:**

In our regional cohort of infants born preterm, ROP, especially severe ROP, was significantly associated with global brain abnormalities at term. Such abnormalities are likely the structural correlates of later functional visual and neurodevelopmental impairments commonly attributed to ROP diagnosis.

Retinopathy of prematurity (ROP) is an abnormal vascular proliferation in the retina during fetal/neonatal development which can lead to visual impairment and blindness.^1^ In the United States, ROP affects 16% of infants born at gestational ages (GAs) of <32 weeks and is the leading cause of treatable blindness in infants born preterm. Its prevalence has been on the rise due to improving survival rates of infants born extremely preterm.^2,3^ Furthermore, infants with severe ROP or ROP requiring treatment display adverse neurodevelopment.^4,5^ The strength of association is on par with other known risk factors for neurodevelopmental impairment (NDI), including bronchopulmonary dysplasia and white matter injury on cranial ultrasound.^6^ However, the reason for this relationship is less clear. Examining the association between ROP and early brain abnormalities, especially those predictive of NDIs, may help clarify this relationship.

Some studies have already established associations between ROP and isolated or regional brain abnormalities on magnetic resonance imaging (MRI) that may provide insight into the relationship between ROP and NDI.^7–10^ For example, ROP was identified as an antecedent for abnormalities in cortical maturation, including reduced gyration and surface area.^9^ Furthermore, ROP has been associated with an increased volume of diffuse white matter abnormality at term.^10^ However, these focal measures do not capture a complete understanding of brain abnormalities that comprise the encephalopathy of prematurity.^11,12^ Advances in neuroimaging of infants born very preterm can now sensitively and comprehensively identify the development of early brain injury by term-equivalent age.

In the present study, we assembled a regional cohort of infants born very preterm to explore the relationship between ROP and brain abnormalities on structural MRI at term-equivalent age using a validated MRI scoring system that comprehensively evaluates the encephalopathy of prematurity, both globally and regionally, using qualitative and quantitative brain measures shown to be predictive of long-term neurodevelopment in infants born preterm.^11^

## Methods

We prospectively recruited a regional cohort of 395 infants born preterm between September 2016 and November 2019. The birth GAs ranged from 23 and 32^6,7^ weeks from 5 neonatal intensive care units (NICUs): Cincinnati Children’s Hospital Medical Center (level IV NICU), University of Cincinnati Medical Center (level III NICU), Good Samaritan Hospital (level III community NICU), St. Elizabeth Healthcare (level III community NICU), and Kettering Health - Main Campus (level III community NICU). Infants were excluded if they had known chromosomal or congenital abnormalities affecting the central nervous system, cyanotic heart disease, or if they were still hospitalized and mechanically ventilated on more than 50% supplemental oxygen at 44 weeks postmenstrual age (PMA). This study was approved by the Cincinnati Children’s Hospital Institutional Review Board. Informed consent for MRI was obtained from all families once infants were clinically stable after 32 weeks PMA. Baseline neonatal and maternal characteristics were prospectively collected using a priori definitions for clinical variables as previously described.^10^

All infants underwent routine ROP screening per guidelines outlined in the International Classification of ROP, third edition^13^ beginning at 31–32 weeks PMA or 5–6 weeks of postnatal age (whichever came later). Patients were classified as having no ROP, any ROP (stages 1–5), or severe ROP (stages 3–5 ROP or type I ROP, a severe form that requires treatment per the International Classification of ROP).^14,15^

All infants underwent brain imaging at 39–44 weeks PMA at Cincinnati Children’s Hospital using a 3T Philips Ingenia MRI scanner with a 32-channel head coil, as previously described.^16^ Brain abnormality burden was quantified by a single, masked pediatric neuroradiologist using the Kidokoro global brain abnormality score.^16^ The scale is based on a composite score that sums abnormalities in 4 brain subcomponents: cortical gray matter (corticalGM), cerebral white matter, deep gray matter (GM), and the cerebellum, graded for severity on a scale of zero to 4. CorticalGM abnormality is graded for each of the following variables: signal abnormality, delayed gyration, and dilated extracerebral cerebrospinal fluid space. Cerebral white matter abnormality is graded for each of the following variables: cystic degeneration, focal signal abnormalities, delayed myelination, thinning of the corpus callosum, dilated lateral ventricles, and reduction of white matter volume. Deep GM and cerebellar abnormalities grading are based on signal abnormality and volume reduction. The raw score of each brain subcomponent abnormality is summed to generate each brain region’s abnormality score. Total cerebral white matter severity scoring is as follows: none (total score, 0–2), mild (total score, 3–4), moderate (total score, 5–6), or severe (total score, ≥7). For total corticalGM, deep GM, and cerebellar scores, the severity score for each is as follows: none (total score, 0), mild (total score, 1), moderate (total score, 2), and severe (total score ≥3). All the brain region scores are further summed to provide a global brain abnormality score.

The Kidokoro scoring system was created to weigh regional brain abnormalities characteristic of the encephalopathy of prematurity that were hypothesized to impair long-term neurodevelopment.^11,12^ For instance, due to the complex nature of cerebral white matter abnormalities, more variables are evaluated in the scoring system resulting in a higher score if significant abnormalities exist in all the variables. The scoring system includes both qualitative and quantitative assessments of brain injury/maturational abnormalities, including quantitative measures of each region: cortical gray (interhemispheric space), subcortical gray (volume of subcorticalGM), cerebellum (transcerebellar diameter), and white matter maturation/development (width lateral ventricles; corpus callosum thickness; biparietal width).^11,16^ When summed up, this ordinal scale, which ranges from 0 to 40, is predictive of standardized measures of neurodevelopment, including motor, language, cognitive, reading, and math scores at ages 2, 7, and 10 years of age in children born very or extremely preterm.^17–19^ Further, these prognostic studies demonstrate a dose-dependent relationship between higher Kidokoro scores and greater severity in neurodevelopmental scores and impairments. Kidokoro et al also offered a categorical severity threshold for the global brain abnormality as follows: normal (total score, 0–3), mild (total score, 4–7), moderate (total score, 8–11), and severe (total score ≥12) that has also been associated with long-term neurodevelopment.^11^

Possible confounding variables associated with ROP and brain abnormalities were identified a priori based on a literature review. These included moderate to severe acute histologic chorioamnionitis using the Redline et al criteria,^20^ hypertensive disorders of pregnancy (chronic and/or pregnancy-induced hypertension, including pre-eclampsia), antenatal maternal cigarette smoking, and GA. In addition, we included the following neonatal comorbidities in our models: white matter injury/abnormalities on early (<1 month of age) head ultrasound, defined as the presence of one or more of the following: grade 3 intraventricular hemorrhage, periventricular hemorrhagic infarction, cystic periventricular leukomalacia, or other types of white matter injury; small for GA, defined as birth weight <10th for GA; necrotizing enterocolitis (stage II or higher) or spontaneous intestinal perforation; and/or postnatal (early or late onset) sepsis.

### Statistical Analysis

SciPy, Pandas, and NumPy open-source Python 3.7 libraries as well as R 4.3.1 were used for statistical analyses. We conducted data imputation for patients with missing data points using k-nearest neighbor in R.^21^ The remaining statistical analyses were run in Python. After confirming normality of the data, the prevalence of neonatal and maternal risk factors was compared between the no ROP and any ROP groups using chi square tests for categorical variables and the Student’s *t*-test for continuous variables. We developed 2 multivariable linear regression models incorporating confounders for any ROP (primary exposure) and severe ROP (secondary) groups to investigate each group’s relationship with global brain abnormality score (primary outcome). Because ROP primarily affects infants born at <30 weeks GA, we repeated these 2 analyses restricted to the 210 infants in our cohort born at <30 weeks GA. We ran a sensitivity analysis to determine whether infant sex and outborn status significantly influenced (>10% change in the ROP beta coefficient) the relationship between ROP and severe ROP to global brain abnormality score. In additional secondary analyses, we used multivariable linear regression to evaluate the relationship between any ROP as well as severe ROP and the 4 subcomponents of the global brain score: deep GM score, total white matter score, cortical GM score, and cerebellar score (cerebellar).^11^

## Results

Of 495 infants consented in the NICU between 32 weeks and 34 weeks PMA, 395 infants completed the MRI study successfully. There were no deaths between consent and MRI appointments. Baseline neonatal and maternal characteristics of the cohort are summarized in Table I. In our cohort, 261 (66.1%) infants had no ROP, 134 (33.9%) infants had any stage of ROP, and 19 (4.8%) infants had severe ROP. Of the infants with severe ROP, 3 received retinal ablation (laser and/or cryotherapy), 6 received an anti-vascular endothelial growth factor (VEGF) drug, and 1 received timolol eye drops. No patients had scleral buckle or vitrectomy performed. Two of the patients received ROP therapy after 120 days, one of which received anti-VEGF therapy and the other received timolol eye drops due to the parents refusing anti-VEGF.

### ROP and Global Brain Abnormality Score

Multivariable models showed that the presence of any stage of ROP was associated with a 1.5- point increase in global brain abnormality score (*β* = 1.5, 95% CI: 0.3–2.8, P = .018; Table II). The presence of severe ROP was associated with a 2.3-point increase in brain abnormality score (*β* = 2.3, 95% CI: 0.1–4.5, P < .001; Table II). In secondary multivariable analyses restricted to infants born at <30 weeks GA (n = 210), any ROP was no longer significantly associated with global brain abnormalities (*β* = 1.1, 95% CI: −0.6 to 2.7, P = .198) but severe ROP remained significant (*β* = 2.6, 95% CI: 0.1–5.0, P = .039). In sensitivity analyses, the addition of sex or outborn status to the above model did not meaningfully affect (<10% change in *β*) the relationships between any ROP or severe ROP and global brain abnormality scores (Table III).

### Association Between ROP and Subcomponents of the Global Brain Abnormality Scores

In secondary multivariable analyses, both any ROP and severe ROP exhibited a significant relationship with deepGM (P < .001 and P = .001, respectively) and cerebellar (P = .017 and P = .012, respectively) subcomponent scores (Tables IV and V). The presence of any stage of ROP was associated with a 0.7-point increase in deepGM score and a 0.5-point increase in cerebellar score on MRI at term-equivalent age. Severe ROP was associated with a 0.9-point increase in deepGM score and a 0.9-point increase in cerebellar score. Neither any ROP nor severe ROP were significantly associated with total white matter or corticalGM scores (Tables IV and V).

## Discussion

In a prospective regional cohort of infants born preterm born at ≤32 weeks GA, we demonstrated an association between ROP and structural brain abnormalities on MRI at term-equivalent age. These early associations between ROP and brain abnormalities at term expand our understanding that the relationship between ROP and adverse neurodevelopmental outcomes may be due to common pathophysiologic mechanisms. This association was even stronger in infants with severe ROP, further reinforcing a potential common pathway between one or more antecedents and concurrent development of ROP and perinatal brain abnormalities. These significant relationships were maintained in multivariable linear regression analyses controlled for several potential confounding variables indicating that ROP was independently associated with brain abnormalities. The association between severe ROP and global brain abnormalities held true in the higher risk subgroup of infants born at <30 weeks GA. In secondary analyses, the association of ROP and the global brain abnormality score was correlated with the brain’s deepGM and cerebellum and not the white matter or corticalGM.

The current literature reports mixed findings for the association between ROP and NDI. Multiple large cohort studies have determined that severe ROP and type I ROP requiring treatment (including anti-VEGF therapy or surgery) are associated with NDI at 18–30 months of age, whereas milder forms of ROP are not.^5,22^ On the other hand, a recent meta-analysis identified that any severity of ROP is associated with NDI diagnosed at 18–48 months of age.^23^ However, the assessment of bias in the meta-analysis determined that the certainty of evidence was “very low”.^23^ It is also unclear whether the mechanism of association between ROP and NDI is mediated solely by injury to retinal development that impairs vision-associated learning or whether a root insult(s) (eg, inflammation) antecedes development of both ROP and brain abnormalities.^24^

The mechanism of association between ROP and NDI can perhaps be better understood by determining ROP’s relationship to a comprehensive assessment of structural brain injuries/maturational abnormalities at term. There is limited prior evidence associating ROP with global neuroimaging changes. For example, in a large cohort of infants born extremely preterm, severe ROP and perinatal brain injury on ultrasound were significantly associated (OR: 7.93; 95% CI: 3.61–17.4) and each were independent and equally predictive risk factors for bilateral blindness with odds of 8.1–8.4, respectively.^25^ A few studies have identified associations between ROP and focal examination of brain abnormalities, including cortical maturational changes (including gyration and surface area), diffuse white matter abnormalities, visual pathways, and smaller cerebellar plus brainstem volumes.^9,10,26^ For example, we found significant differences in the macrostructure and microstructure of several white matter tracts that subserve visual function (eg, posterior thalamic radiations, inferior longitudinal fasciculus, superior longitudinal fasciculus, etc.) on diffusion MRI at term in infants born preterm with and without ROP from this same cohort.^27^ These measures only provide a limited scope in understanding brain abnormalities and/or may have measurement systems subject to bias.^11^ In this study, we observed a significant increase in global brain abnormality score in infants born preterm with ROP revealing widespread brain injury and/or immaturity.

The Kidokoro scoring system is a standardized comprehensive scoring system that is predictive of later childhood neurodevelopment, including performance on the Bayley Scales of Infant and Toddler Development as well as long-term IQ and math computation.^19^ Anderson et al (2017) reported that a one-point increase in the global brain and deepGM abnormality scores on MRI at term were predictive of 1.4- and 4.8-point decreases in IQ scores, respectively and 1.6- and 5.5-point decrements in math computation scores respectively at age 7, independent of known perinatal and social predictors.^19^ For children with severe ROP exposure in our cohort who had a 2.3 and 0.9 independent increase in global brain and deepGM abnormality scores, this could potentially equate to decrements of 3.2–4.3 points in IQ and 3.7–4.9 points in math scores at 7 years of age, independent of known perinatal and social predictors.

Our study confirmed that ROP is associated with widespread brain injury/development at term-equivalent age, independent of perinatal/neonatal confounding factors. We also confirmed that greater severity of ROP is associated with more significant structural brain abnormalities, particularly in the deepGM and cerebellum. Our findings associating ROP exposure to abnormalities in these 2 regions is consistent with prior imaging studies in infants born preterm. Several studies have linked isolated cerebellar hemorrhage and/or volumetric abnormalities with long-term visual perception abnormalities.^28,29^ Investigators have also identified an association between basal ganglia damage and impaired visual function where large occipital lobe lesions were not associated with visual impairment unless concomitant basal ganglia damage was also present.^30^ On functional MRI, adolescents born preterm have been shown to activate several brain regions, including the caudate and cerebellum, more robustly during a visual perception task as compared with adolescents born at term.^31^ We propose 3 pathophysiologic etiologies that are associated with premature birth that may also be acting as causal mechanisms to the development of both ROP and brain abnormalities: hyperoxia, insulin-like growth factor 1 (IGF-1) deficiency, and inflammation.^24^

Conditions ex-utero are relatively hyperoxic compared with in utero as uterine arterial oxygen saturation lies around 70%. Both retinal and brain development thrive in the lower oxygen conditions in-utero. Premature delivery exposes infants to higher oxygen levels before the completion of brain and retinal development. In the retina, hyperoxia ex-utero initially arrests vascular development. This leads to a zone of relative ischemia in the peripheral retina that induces the production of vascular growth factors such as VEGF, stimulates abnormal vascular proliferation, and potentially progresses to ROP.^32^ The necessary use of supplemental oxygen in neonates for resuscitation and postnatal survival can further exacerbate these hyperoxia effects. In the developing brain, hyperoxia causes gray and white matter injury via oxidative stress mechanisms that induce apoptosis in oligodendrocytes and neurons. Furthermore, chronic hyperoxia has been shown to specifically decrease cerebellar and hippocampal volumes in animal models offering a potential explanation for the association between ROP and cerebellar and deep gray matter structures.^33^

A second potential causal pathway involves IGF-1 deficiency. IGF-1 is a crucial regulator of fetal growth and organ development. Infants born preterm have very low IGF-1 serum levels that remain low throughout perinatal development.^34^ IGF-1 plays a role in retinal development by working with VEGF to stimulate retinal vasculature. A deficiency of IGF-1 during this period, even with the presence of VEGF, has been shown to prevent normal retinal vascular development and results in ROP.^32^ IGF-1 deficiency has also been shown to impair brain development in all brain regions (corticalGM, white matter, deep GM, and the cerebellum) in infants born preterm leading to lower brain volumes suggesting IGF-1 could also play a role in the association between ROP and brain abnormalities.^35^

A last potential causal pathway involves proinflammatory cytokines. Proinflammatory cytokines contribute to ROP by upregulating VEGF signaling, thus leading to abnormal retinal vascular proliferation.^36^ When inflammatory proteins such as tumor necrosis factor alpha, tumor necrosis factor receptor 1 and 2, interleukins 6 and 8, serum amyloid A, c-reactive peptide, intercellular adhesion molecule 1, and/or myeloperoxidase present at high levels in the first postnatal month, there is an increased risk of ROP development.^37^ In the perinatal period, inflammation also damages the developing brain, particularly oligodendrocyte precursors, causing white matter regions to sustain the most significant damage.^38^ It is notable in our study that severe ROP was not associated with white matter damage, potentially decreasing the likelihood of this explanation. Elevated levels of erythropoietin, which are typically present with inflammation, have also been shown to be associated with both ROP development and neurodevelopmental delay.^24^

We observed a significant increase in widespread brain injury and/or immaturity at term-equivalent age in infants born preterm with ROP. Our study findings clearly demonstrate that the association between ROP and NDI likely results from more than the known antecedents of retinal damage or isolated cerebral visual pathway injury that can adversely affect neurodevelopment. Thus, the most plausible hypothesis to explain this association is a common root insult that is likely causal in the development of ROP and perinatal brain abnormalities, especially in the deepGM and cerebellum, and ultimately NDI. Clinically, our findings strengthen the importance of identifying the underlying causes of ROP and perinatal brain abnormalities, especially those beyond isolated white matter injury to the visual tracts.

Our study has several strengths. We studied a relatively large and regionally representative cohort from 5 academic and community NICUs. In addition, we used a comprehensive approach to quantify brain abnormalities using standardized and validated approaches (the Kidokoro scoring system) with raters that were blinded to infant baseline characteristics and a single neuroradiologist who scored all brain MRI scans with high intrarater reliability, thus decreasing measurement error, as previously demonstrated.^39^ Our study also has several limitations. Although we incorporated the most common morbidities and confounders among the preterm population into our models, our study is limited in that some residual confounding is still possible. In addition, using a linear regression model is limited in that the relationship between ROP and brain abnormalities may not be perfectly linear. Lastly, although we and others have established the validity of the global brain abnormality score to predict long-term neurodevelopmental outcomes, we do not yet have school-age neurodevelopmental outcomes for this cohort.

In conclusion, we identified ROP as a correlate for widespread brain abnormalities by term-equivalent age in a regional cohort of infants born preterm. Such abnormalities are likely the structural correlates of later functional visual and NDIs commonly associated with ROP diagnosis. An important next step will be to determine how much of the link between ROP and NDI is indirectly mediated via brain abnormalities at term-equivalent age and how much, if any, is a direct causal link.

## Figures and Tables

**Table I.** Baseline perinatal and neonatal maternal and infant characteristics of a regional cohort of infants born at ≤32 weeks gestational age with and without retinopathy of prematurity

Characteristic	No ROP (n = 261)	Any ROP (n = 134)	*P* value
Moderate-severe histologic chorioamnionitis*	27 (10%)	30 (22%)	*.002*
Hypertensive disorders of pregnancy	116 (44%)	54 (40%)	.50
Antenatal maternal cigarette smoking	29 (11%)	21 (16%)	.26
Gestational age (median weeks, IQR)	31,3	27, 3	<*.001*
< 28 weeks	14 (5%)	81 (60%)	<*.001*
28–29 weeks	58 (22%)	39 (29%)	*.132*
30–31 weeks	97 (37%)	10 (7%)	<*.001*
32–32^6/7^ weeks	92 (35%)	4 (3%)	<*.001*
Small for gestational age (<10th percentile)	14 (5%)	13 (10%)	.16
White matter injury on early head ultrasound^†^	13 (5%)	24 (18%)	<*.001*
Postnatal sepsis	14 (5%)	30 (22%)	<*.001*
Necrotizing enterocolitis (NEC; stage II or higher) or spontaneous intestinal perforation (SIP)	8 (3%)	14 (10%)	*.005*

All values are n (%) unless otherwise indicated. P values <.05 are highlighted in italics.

* Forty-two patients had missing data for moderate-severe histologic chorioamnionitis.

† White matter injury/abnormality including grade 3 intraventricular hemorrhage, periventricular hemorrhagic infarction, cystic periventricular leukomalacia and/or other white matter injury on early head ultrasound before 1 month of age.

**Table II.** Multivariable regression results for the association between any ROP or severe ROP, adjusting for potential confounding variables in the first column, and global brain abnormality score on magnetic resonance imaging at term-equivalent age

	Any ROP		Severe ROP	
Characteristic	Coefficient (95% CI)	*P* value	Coefficient (95% CI)	*P* value
Any ROP	1.5 (0.3–2.8)	*.018*		
Severe ROP			2.3 (0.1–4.5)	<*.001*
Moderate to severe chorioamnionitis	1.0 (−0.3 to 2.3)	.115	0.8 (−0.5 to 2.1)	.249
Hypertensive disorders of pregnancy	0.7 (−0.2 to 1.6)	.107	0.8 (−0.1 to 1.7)	.071
Antenatal maternal cigarette smoking	2.4 (1.1–3.7)	<*.001*	2.3 (1.0–3.6)	*.001*
Gestational age (continuous variable)	−0.3 (−0.5 to −0.02)	*.038*	−0.4 (−0.6 to −0.2)	<*.001*
Small for gestational age	3.9 (2.1–5.6)	<*.001*	4.3 (2.6–6.0)	<*.001*
White matter injury	5.1 (3.6–6.7)	<*.001*	5.2 (3.7–6.7)	<*.001*
Postnatal sepsis	1.4 (−0.1 to 2.9)	.065	1.4 (−0.1 to 2.9)	.063
NEC/SIP	3.7 (1.7–5.6)	<*.001*	3.4 (1.5–5.4)	*.001*

P values <.05 are italicized.

**Table III.** Sensitivity analysis adding infant sex and outborn status to the multivariable regression analysis of the association between any ROP or severe ROP, adjusting for all potential confounding variables (first column), and global brain abnormality score on magnetic resonance imaging at term-equivalent age

	Global brain abnormality score (any ROP)		Global brain abnormality score (severe ROP)	
Characteristic	Coefficient (95% CI)	*P* value	Coeffficient (95% CI)	*P* value
Any ROP	1.5 (0.2–2.8)	*.020*		
Severe ROP			2.1 (−0.1 to 4.3)	.063
Moderate to severe chorioamnionitis	1.2 (−0.2 to 2.5)	.081	0.9 (−0.4 to 2.2)	.188
Hypertensive disorders of pregnancy	0.8 (−0.1 to 1.7)	.063	0.9 (0.0–1.8)	*.045*
Antenatal maternal cigarette smoking	2.3 (1.0–3.6)	*.001*	2.2 (0.9–3.6)	*.001*
Gestational age	−0.3 (−0.5 to 0.0)	*.036*	−0.4 (−0.6 to −0.2)	<*.001*
Small for gestational age	3.9 (2.2–5.6)	<*.001*	4.3 (2.6–6.0)	<*.001*
White matter injury	5. (3.4–6.5)	<*.001*	5.1 (3.5–6.6)	<*.001*
Postnatal sepsis	1.4 (−0.1 to 2.9)	.064	1.4 (−0.1 to 2.9)	.063
NEC/SIP	3.4 (1.5–5.4)	*.001*	3.3 (1.3–5.2)	*.001*
Infant sex (female)	0.1 (−0.7 to 1.0)	.777	0.2 (−0.7 to 1.0)	.696
Outborn status	1.45 (−0.4 to 3.3)	.120	1.3 (−0.6 to 3.2)	.168

P values <.05 are italicized.

**Table IV.** Multivariable regression results for the association between any ROP, adjusting for all potential confounding variables (first column), and subcomponents of global brain abnormality score on magnetic resonance imaging at term-equivalent age in a cohort of infants born ≤32 weeks gestational age

	Deep nuclear gray matter		Total white matter		Cortical gray matter		Cerebellar	
Characteristic	Coeffficient (95% CI)	*P* value	Coeffficient (95% CI)	*P* value	Coeffficient (95% CI)	*P* value	Coeffficient (95% CI)	*P* value
Any ROP	0.7 (0.4–1.0)	<*.001*	0.3 (−0.4 to 1.1)	.409	0.0 (−0.4 to 0.4)	.929	0.5 (0.1–0.9)	*.017*
Moderate to severe chorioamnionitis	0.2 (−0.1 to 0.5)	.258	0.3 (−0.5 to 1.1)	.433	0.3 (−0.1 to 0.7)	.115	0.3 (−0.2 to 0.7)	.252
Hypertensive disorders of pregnancy	0.1 (−0.1 to 0.3)	.320	0.4 (−0.1 to 1.0)	.098	0.1 (−0.2 to 0.4)	.462	0.1 (−0.2 to 0.4)	.585
Antenatal maternal cigarette smoking	0.4 (0.1–0.7)	*.013*	1.3 (0.6–2.1)	*.001*	0.4 (0.1–0.8)	*.023*	0.2 (−0.2 to 0.6)	.386
Gestational age	0.0 (0.0–0.1)	.613	−0.2 (−0.3 to 0.0)	*.041*	0.0 (−0.1 to 0.0)	.419	−0.1 (−0.2 to 0.0)	.120
Small for gestational age	1.0 (0.6–1.4)	<*.001*	1.9 (0.9–2.9)	<*.001*	0.1 (−0.4 to 0.6)	.817	0.9 (0.3–1.4)	*.004*
White matter injury	0.9 (0.5–1.3)	<*.001*	3.0 (2.1–3.9)	<*.001*	0.3 (−0.2 to 0.7)	.258	1.0 (0.5–1.5)	<*.001*
Postnatal sepsis	0.5 (0.1–0.8)	*.010*	0.4 (−0.5 to 1.3)	.403	−0.4 (−0.8 to 0.1)	.100	0.9 (0.4–1.4)	<*.001*
NEC/SIP	1.0 (0.6–1.5)	<*.001*	0.5 (−0.6 to 1.7)	.349	0.3 (−0.3 to 0.8)	.323	1.8 (1.2–2.4)	<*.001*

P values <.05 are italicized.

**Table V.** Multivariable regression results for the association between severe ROP, adjusting for all potential confounding variables (first column), and subcomponents of global brain abnormality score on magnetic resonance imaging at term-equivalent age

	Deep nuclear gray matter		Total white matter		Cortical gray matter		Cerebellar	
Characteristic	Coeffficient (95% CI)	*P* value	Coeffficient (95% CI)	*P* value	Coeffficient (95% CI)	*P* value	Coeffficient (95% CI)	*P* value
Severe ROP	0.9 (0.4–1.4)	*.001*	0.6 (−0.7 to 1.9)	.336	−0.2 (−0.8 to 0.5)	.593	0.9 (0.2–1.7)	*.012*
Moderate to severe chorioamnionitis	0.1 (−0.3 to 0.4)	.668	0.2 (−0.5 to 1.0)	.547	0.3 (−0.1 to 0.7)	.102	0.2 (−0.3 to 0.6)	.507
Hypertensive disorders of pregnancy	0.2 (−0.1 to 0.4)	.188	0.5 (−0.1 to 1.0)	.082	0.1 (−0.2 to 0.3)	.486	0.1 (−0.2 to 0.4)	.434
Antenatal maternal cigarette smoking	0.4 (0.1–0.7)	*.019*	1.3 (0.6–2.1)	*.001*	0.4 (0.1–0.8)	*.022*	0.2 (0.3–0.6)	.427
Gestational age	−0.1 (−0.1 to 0.0)	*.001*	−0.2 (−0.3 to −0.1)	*.003*	0.0 (−0.1 to 0.0)	.220	−0.1 (−0.2 to −0.0)	*.001*
Small for gestational age	1.2 (0.8–1.6)	<*.001*	2.0 (1.0–3.0)	<*.001*	0.1 (−0.4 to 0.6)	.821	1.0 (0.4–1.6)	*.001*
White matter injury	0.9 (0.5–1.3)	<*.001*	3.0 (2.1–3.9)	<*.001*	0.3 (−0.2 to 0.7)	.244	1.0 (0.5–1.5)	<*.001*
Postnatal sepsis	0.5 (0.1–0.8)	*.010*	0.4 (−0.5 to 1.3)	.400	−0.4 (−0.8 to 0.1)	.100	0.9 (0.4–1.4)	<*.001*
NEC/SIP	1.0 (0.5–1.5)	<*.001*	0.5 (−0.7 to 1.6)	.415	0.3 (−0.3 to 0.9)	.287	1.7 (1.0–2.2)	<*.001*

P values <.05 are italicized.

## Data Availability

Data sharing statement available at www.jpeds.com.

## Abbreviations

CorticalGM: Cortical gray matter
DeepGM: Deep nuclear gray matter
GA: Gestational age
IGF-1: Insulin-like growth factor 1
MRI: Magnetic resonance imaging
NDI: Neurodevelopmental impairment
NICU: Neonatal intensive care unit
PMA: Postmenstrual age
ROP: Retinopathy of prematurity
VEGF: Vascular endothelial growth factor
